# No requirement of TRPV1 in long-term potentiation or long-term depression in the anterior cingulate cortex

**DOI:** 10.1186/1756-6606-7-27

**Published:** 2014-04-05

**Authors:** Ming-Gang Liu, Min Zhuo

**Affiliations:** 1 Center for Neuron and Disease, Frontier Institute of Science and Technology, Xi’an Jiaotong University, Xi’an 710049, China; 2 Department of Physiology, Faculty of Medicine, University of Toronto, 1 King's College Circle, Toronto, Ontario M5S 1A8, Canada

**Keywords:** Anterior cingulate cortex, Long-term potentiation, Long-term depression, Multi-electrode array, Transient receptor potential vanilloid type 1, Chronic pain

## Abstract

One major interest in the study of transient receptor potential vanilloid type 1 (TRPV1) in sensory system is that it may serve as a drug target for treating chronic pain. While the roles of TRPV1 in peripheral nociception and sensitization have been well documented, less is known about its contribution to pain-related cortical plasticity. Here, we used 64 multi-electrode array recording to examine the potential role of TRPV1 in two major forms of synaptic plasticity, long-term potentiation (LTP) and long-term depression (LTD), in the anterior cingulate cortex (ACC). We found that pharmacological blockade of TRPV1 with either [(E)-3-(4-t-Butylphenyl)-N-(2,3-dihydrobenzo[b][1,4]dioxin-6-yl)acrylamide] (AMG9810, 10 μM) or N-(3-methoxyphenyl)-4-chlorocinnamide (SB366791, 20 μM) failed to affect LTP induced by strong theta burst stimulation in the ACC of adult mice. Similarly, neither AMG9810 nor SB366791 blocked the cingulate LTD induced by low-frequency stimulation. Analysis of the results from different layers of the ACC obtained the same conclusions. Spatial distribution of LTP or LTD-showing channels among the ACC network was also unaltered by the TRPV1 antagonists. Since cortical LTP and LTD in the ACC play critical roles in chronic pain triggered by inflammation or nerve injury, our findings suggest that TRPV1 may not be a viable target for treating chronic pain, especially at the cortical level.

## Introduction

The transient receptor potential vanilloid type 1 (TRPV1) is known to act as a molecular detector for a wide range of physical (>42°C temperature) and chemical (low PH, capsaicin) stimuli in the periphery [1-4]. Pharmacological blockade or genetic deletion of TRPV1 channel can produce significant analgesic effects in acute pain as well as inflammatory pain [5-9]. However, controversy still exists regarding the role of TRPV1 in chronic neuropathic pain. While there are some pharmacological data showing the blockade of neuropathic hypersensitivity by TRPV1 antagonists [10-13], TRPV1 knockout mice exhibited no change in mechanical allodynia or thermal hyperalgesia following peripheral nerve injury [5,7].

In addition to its distribution in primary sensory neurons, TRPV1 has also been found in many regions of the brain [14-19], also see Table 1]. Neurophysiological and pharmacological studies demonstrate that TRPV1 is important for certain forms of synaptic plasticity, such as long-term potentiation (LTP) and long-term depression (LTD), in different central synapses [20], also see Table 2]. For example, LTP in the hippocampus is impaired in the TRPV1-deficient mice [21-23]. Moreover, TRPV1 receptors can mediate a presynaptic form of LTD in the hippocampus [24] and a postsynaptic form of LTD in the dentate gyrus [25] and nucleus accumbens [26].

**Table 1:** Summary of previous studies on the distribution of TRPV1 in the brain

**Species**	**Methods**	**Brain regions**									**Reference**
		**OB**	**CC**	**HP**	**AMY**	**TH**	**HT**	**PAG**	**LC**	**CB**	
Rat and human	ISH, IHC and RT-PCR	Yes	Yes	Yes	Yes	Yes	Yes	Yes	Yes	Yes	[14]
Mouse	IHC	NT	NT	Yes	NT	Yes	Yes	Yes	NT	Yes	[15]
Rat	[^3^H]RTX binding assay	Yes	Yes	Yes	Yes	Yes	Yes	Yes	NT	Yes	[17]
Rat	RT-PCR	Yes	Yes	Yes	NT	Yes	Yes	Yes	NT	Yes	[18]
Rat	RPA	Yes	Yes	Yes	Yes	NT	No	NT	NT	Yes	[19]
Rat	WB, IHC and IEM	Yes	Yes	Yes	NT	Yes	NT	Yes	Yes	Yes	[27]
Rat and human	[^3^H]RTX binding assay	NT	No	NT	NT	Yes	Yes	Np	Yes	No	[28]
Monkey	[^3^H]RTX binding assay	NT	Yes	NT	NT	Yes	Yes	Yes	Yes	Yes	[29]
Rat	IHC	NT	Yes	NT	NT	NT	NT	NT	NT	NT	[30]

Notes: AMY, amygdala; CB, cerebellum; CC, cerebral cortex; HP, hippocampus; HT, hypothalamus; IEM, immonoelectronmicroscopy; IHC, immunohistochemistry; ISH, in situ hybridization; LC, locus coeruleus; NT, not tested; OB, olfactory bulb; PAG, periaqueductal gray; RPA, ribonuclease protection assay; RT-PCR, reverse transcription-polymerase chain reaction; RTX, resiniferatoxin; TH, thalamus; WB, western blot.

**Table 2:** Summary of previous studies on TRPV1 and synaptic plasticity in the brain

**Brain area**	**Species**	**Method**	**Plasticity type**	**Key findings**	**Reference**
Hippocampus	Mouse	fEPSP	LTP	Reduced LTP in the CA1 region in TRPV1-deficient mice	[21]
CA1 region					
Hippocampus	Rat	fEPSP	LTP and LTD	TRPV1 agonists facilitated LTP but suppressed LTD induction and thus antagonized the stress effect on synaptic plasticity in the CA1 region	[22]
CA1 region					
Hippocampus	Rat	fEPSP	LTP and LTD	TRPV1 activation facilitates LTP induction via disinhibition, reduces DHPG-induced acute depression, but does not affect LFS-induced LTD in CA1	[31]
CA1 region					
Hippocampus	Rat	EPSC	LTD	TRPV1 channels activated by 12-(S)-HPETE mediate a presynaptic form of LTD in the excitatory synapses on CA1 interneurons	[24]
CA1 region					
Hippocampus	Rat	EPSC	LTD	Calcineurin is required for TRPV1-induced LTD in the CA1 interneurons	[32]
CA1 region					
Hippocampus	Mouse	fEPSP and EPSC	LTP and LTD	Loss of interneuron LTD and attenuated pyramidal cell LTP in the TRPV1 knockout mice	[23]
CA1 region					
Dentate gyrus	Rat and mouse	EPSC	LTD	TRNPV1 channels activated by anandamide mediate a postsynaptic form of LTD in dentate granular cells	[25]
Superior colliculus	Mouse	fEPSP	LTD	TRPV1 channels mediate LTD in the developing superior colliculus	[33]
Nucleus accumbens	Mouse	EPSC	LTD	TRPV1 channels activated by endocannabinoid mediate a postsynaptic form of LTD in medium spiny neurons of the indirect pathway	[26]
BNST and dorsal striatum	Rat	fEPSP and EPSC	LTD	TRPV1 channels activated by anandamide mediate LTD in the BNST but not dorsal striatum	[34]
Lateral amygdala	Mouse	fEPSP	LTP	TRPV1 agonist affected the LTP in a way that is dependent on the anesthetic	[35]
ACC	Mouse	fEPSP	LTP and LTD	TRPV1 antagonosts had no effect on cingulate LTP or LTD induction	The present study

Notes: 12-(S)-HPETE, 12(S)-hydroperoxyeicosa-5Z, 8Z, 10E, 14Z-tetraenoic acid; ACC, anterior cingulate cortex; BNST, bed nucleus of the stria terminalis; DHPG, (RS)-3,5-dihydroxy phenylglycine; EPSC, excitatory postsynaptic current; fEPSP, field excitatory postsynaptic potential; LFS, low-frequency stimulation; LTD, long-term depression; LTP, long-term potentiation; TRPV1, transient receptor potential vanilloid type 1.

It is believed that sensory plasticity taking place in different levels of the central nervous system is important for chronic pain [36-39]. In addition to the sensitization occurring at the level of periphery and spinal cord [40,41], recent studies demonstrate that cortical plasticity might also contribute critically to chronic pain [for reviews, see [38,39,42]. Peripheral tissue or nerve injury triggers LTP-like enhancement of the excitatory synaptic transmission in the anterior cingulate cortex (ACC) [43-47]. Drugs that block the induction or erasing the maintenance of cingulate LTP produce analgesic effects in animal pain models [39,46,48-52]. These plastic changes are in accordance with biochemical studies that reveal upregulation of LTP-related receptors and molecules, such as NMDA receptor, AMPA receptor and synaptic vesicle proteins, in the ACC by various forms of pain [44,51,53-56]. Therefore, cortical plasticity can be used as a novel endpoint measurement for screening and evaluating analgesic compounds in translational pain studies [42].

The TRPV1 channel has increasingly been considered to be a promising therapeutic target for clinical pain treatment, despite reported side effects on body temperature [57-59]. In animal behavioral tests, both TRPV1 receptor agonists and antagonists have been tested for the treatment of inflammatory or neuropathic pain [60,61]. Electrophysiological recordings show the lack of spinal LTP in TRPV1 deficient mice [62]. However, to our knowledge, no report is available on its contribution to pain-related cortical plasticity. Here we used a 64-channel multi-electrode dish (MED64) recording system [63-65] to investigate the effects of two TRPV1 receptor antagonists, (E)-3-(4-t-Butylphenyl)-N-(2,3-dihydrobenzo[b][1,4] dioxin-6-yl)acrylamide (AMG9810) and N-(3-methoxyphenyl)- 4-chlorocinnamide (SB366791), on the induction of LTP and LTD in the ACC. Interestingly, we found that neither AMG9810 nor SB366791 had any effect on the synaptic plasticity in the adult mouse ACC.

## Materials and methods

### Animals

The experiments were carried out on male C57BL/6 mice (7–10 week old, Charles River, Quebec, Canada). All animals were fed in groups of three per cage under standard laboratory conditions (12 h light/12 h dark, temperature 22-26°C, air humidity 55-60%) with *ad libitum* water and mice chow. The experimental procedures were approved by the Institutional Animal Care and Use Committee of The University of Toronto. The number of animals used and their suffering were greatly minimized.

### Drugs

All drugs were purchased from Tocris Cookson (Bristol, UK). Both AMG9810 and SB366791 were dissolved in dimethyl sulfoxide (DMSO) as stock solutions and were diluted to the final desired concentration in the artificial cerebrospinal fluid (ACSF) before immediate use. The selectivity of the two drugs against TRPV1 has been demonstrated previously [66,67]. The concentration of DMSO in the ACSF was maintained at <0.1%. For the LTP experiment, the drugs were applied in a bath solution from 20 min before conditioning stimuli until 20 min after LTP induction. For the LTD experiment, both agents were bath applied 25 min prior to and during the LTD induction. None of the above drugs affected basal synaptic transmission in the ACC.

### Slice preparation

The general procedures for making the ACC slices are similar to those described previously [46,63,68]. Briefly, mice were anesthetized with gaseous isoflurane and decapitated. The whole brain was rapidly removed and immersed into a cold bath of oxygenated (equilibrated with 95% O2 and 5% CO2) ACSF containing (in mM): NaCl 124, KCl 2.5, NaH_2_PO_4_ 1.0, MgSO_4_ 1, CaCl_2_ 2, NaHCO_3_ 25 and glucose 10, pH 7.35-7.45. After cooling for 1–2 min, appropriate portions of the brain were then trimmed and the remaining brain block was glued onto the ice-cold stage of a vibrating tissue slicer (Leika, VT1000S). Then three coronal ACC slices (300 μm) were obtained at the level of corpus callosum connection and transferred to an incubation chamber continuously perfused with oxygenated ACSF at 26°C. Slices were allowed to recover for at least 2 h before any electrophysiological recording was attempted.

### Multi-channel field potential recordings

A commercial 64-channel recording system (MED64, Panasonic Alpha-Med Sciences, Japan) was used for extracellular field potential recordings in this study. Procedures for preparation of the MED64 probe and multi-channel field potential recordings were similar to those described previously [63-65,68,69]. The MED64 probe had an array of 64 planar microelectrodes, each 50 × 50 μm in size, arranged in an 8 × 8 pattern (inter-electrode distance: 150 μm). Before use, the surface of the MED64 probe was treated with 0.1% polyethyleneimine (Sigma) in 25 mM borate buffer (pH 8.4) overnight at room temperature. After incubation, one slice was positioned on the MED64 probe in such a way that the ACC area was entirely covered by the recording dish mounted on the stage of an inverted microscope (CKX41, Olympus). Once the slice was settled, a fine mesh anchor (Warner Instruments, Harvard) was carefully positioned to ensure slice stability during recording. The slice was continuously perfused with oxygenated, fresh ACSF at the rate of 2–3 ml/min with the aid of a peristaltic pump (Minipuls 3, Gilson) throughout the entire experimental period.

After a 10–15 min recovery period, one of the 64 available planar microelectrodes was selected from the 64-switch box for stimulation by visual observation through a charge-coupled device camera (DP70, Olympus) connected to the inverted microscope. For test stimulation, monopolar, biphasic constant current pulses (0.1 ms in duration) generated by the data acquisition software (Mobius, Panasonic Alpha-Med Sciences) were applied to the deep layer (layer V-VI) of the ACC slice at 0.008 Hz. The field excitatory postsynaptic potentials (fEPSPs) evoked at both superficial layer (layer II-III) and deep layer of the ACC were amplified by a 64-channel amplifier, displayed on the monitor screen and stored on the hard disk of a microcomputer for off-line analysis. Baseline synaptic responses were first stabilized for at least 20 min before any conditioning stimulation. For LTP induction, a theta burst stimulation (TBS) protocol (5 bursts at 5 Hz, repeated 5 times at 10 s intervals, four pulses at 100 Hz for each burst) was given at the stimulation intensity which was adjusted to elicit 40-60% of the maximal response [46]. For LTD induction, a classical low-frequency stimulation (LFS) protocol (1 Hz, 900 pulses, with the same intensity as baseline recording) was used as described previously [63,65,69]. After TBS or LFS, the test stimulus was repeatedly delivered once every 2 min for 1–2 h to monitor the time course of LTP or LTD.

### Data analysis

All multi-channel electrophysiological data were analyzed off-line by the MED64 Mobius software. For quantification of the LTP and LTD data, the initial slope of fEPSPs was measured by taking the rising phase between 10% and 90% of the peak response, normalized and presented separately in both superficial and deep layers as a percentage change from the baseline level. The degree of LTP or LTD in each experiment was shown as the value obtained at 2 h or 1 h after TBS or LFS, respectively. For comparison of the LTP/LTD magnitude between different treatments, the averaged value of the last 10 min of recordings was compared statistically. Furthermore, the number of activated channels (over 20% of baseline, i.e. the amplitude goes over −20 μV) vs. LTP-showing (increased by at least 20% of baseline) or LTD-showing (depressed by at least 15% of baseline) channels was counted and expressed as the induction ratio of LTP/LTD (number of LTP/LTD-occurring channels/number of all activated channels × 100%) [64,65,69]. All data are presented as mean ± S.E.M. When necessary, the statistical significance was assessed by two-tailed Student’s t test or Mann–Whitney rank sum test using the SigmaPlot11.0 software. *P* < 0.05 was assumed to be statistically significant.

## Results

### Pharmacological blockade of TRPV1 with AMG9810 does not affect LTP induction in the ACC

**Figure 1:**
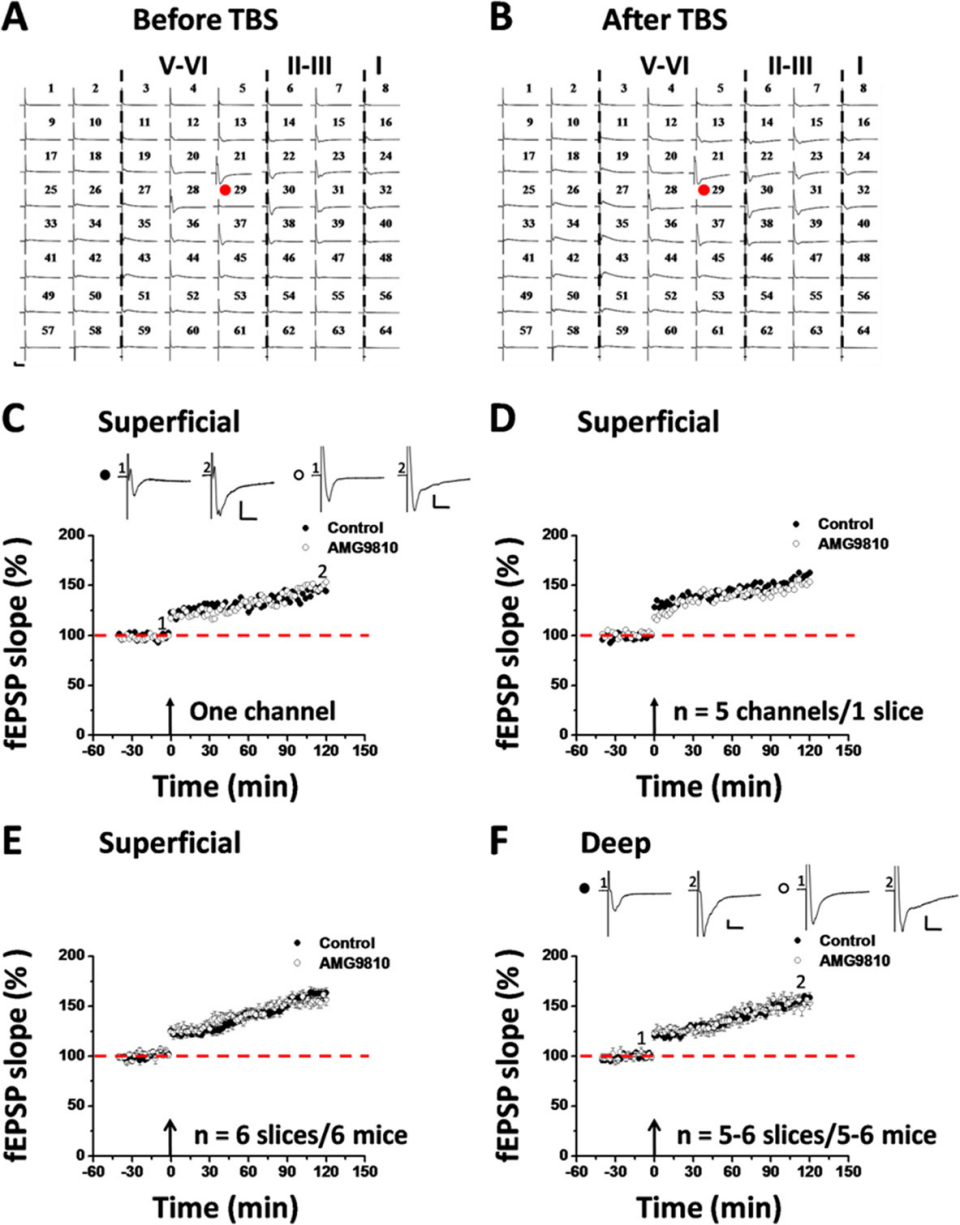
**Pharmacological blockade of TRPV1 activation with AMG9810 does not affect the LTP induction in the ACC. (A and B)** An overview of 64-channel multi-electrode array recordings in one AMG9810-treated ACC slice (**A**: before TBS; **B**: 2 h after TBS). After stabilizing the baseline responses for 20 min, AMG9810 (10 μM) was bath applied from 20 min before till 20 min after delivery of TBS to the deep layer. It could be apparently seen that AMG9810 infusion could not prevent the LTP induction. Red circles indicate the stimulated channel (Ch. 29). Vertical lines demarcate the different layers in the ACC. **(C)** Results of one LTP-showing channel (Ch. 38 and Ch. 30) from the superficial layer of one slice for control and AMG9810-treated group, respectively. **(D)** Summary of averaged data from 5 superficial layer channels of one slice for both control and AMG9810-applied group, respectively. **(E)** Pooled data of the superficial layer of the ACC from 6 slices from 6 mice. **(F)** Pooled data in the deep layer of the ACC (n = 5–6 slices/5-6 mice). TBS delivery results in an enduring synaptic potentiation that lasts for at least 2 h, which is not affected by the presence of AMG9810. Insets in **(C and F)**: representative fEPSP traces at time points indicated by the numbers in the graph. Arrows in **(C-F)** denote the starting point of TBS application. Calibrations in **(A, C and F)**: 100 μV, 10 ms. Error bars in **(E and F)** represent SEM.

The important role of TRPV1 in LTP induction in the hippocampus has been shown in previous work using the genetically modified mice [21-23]. In the present study, we employed a previously-established 64-channel multi-electrode array system [64,65,68,69] to examine whether TRPV1 is equally important for cingulate LTP. After stabilizing the baseline responses for at least 20 min, we bath applied AMG9810 (10 μM), a selective TRPV1 antagonist [67], for 20 min prior to delivery of TBS to the ACC slice. The drug was washed out 20 min after TBS and LTP was monitored for 2 h. One representative 64-channel recording is illustrated in Figure 1A (before TBS) and Figure 1B (2 h after TBS) for the AMG9810-treated group. It could be seen that TBS still induced a long-lasting potentiation of fEPSP within the ACC network. Analysis of one single channel (Ch. 30) in the superficial layer (layer I-III) revealed that the response was potentiated to 153.2% of baseline at 2 h after TBS, with the LTP magnitude being comparable with the control (Ch. 38, 143.8% of baseline; Figure 1C). The averaged data from 5 superficial channels for each condition is plotted in Figure 1D (control vs. AMG9810: 162.8% vs. 153.4% of baseline). Pooled data from 6 slices of 6 mice are presented in Figure 1E (control: 163.4 ± 5.0% of baseline at 2 h after TBS, n = 6 slices/6 mice, *P* < 0.001, Paired t-test; AMG9810: 156.6 ± 5.2% of baseline, n = 6 slices/6 mice, *P* < 0.001, Paired t-test).

Similar results were obtained when analyzing the channels located in the deep layer (layer V-VI) of the ACC. As shown in Figure 1F, infusion of AMG9810 failed to block the LTP induction (control: 158.8 ± 4.8% of baseline at 2 h after TBS, n = 5 slices/5 mice, *P* = 0.002, Paired t-test; AMG9810: 153.6 ± 6.6% of baseline, n = 6 slices/6 mice, *P* < 0.001, Paired t-test). Statistical analysis does not detect any significant difference in the degree of LTP between control and AMG9810-treated group in either superficial layer (*P* = 0.818, Mann–Whitney ran sum test, Figure 1E) or deep layer of the ACC (*P* = 0.335, Unpaired t-test, Figure 1F).

**Figure 2:**
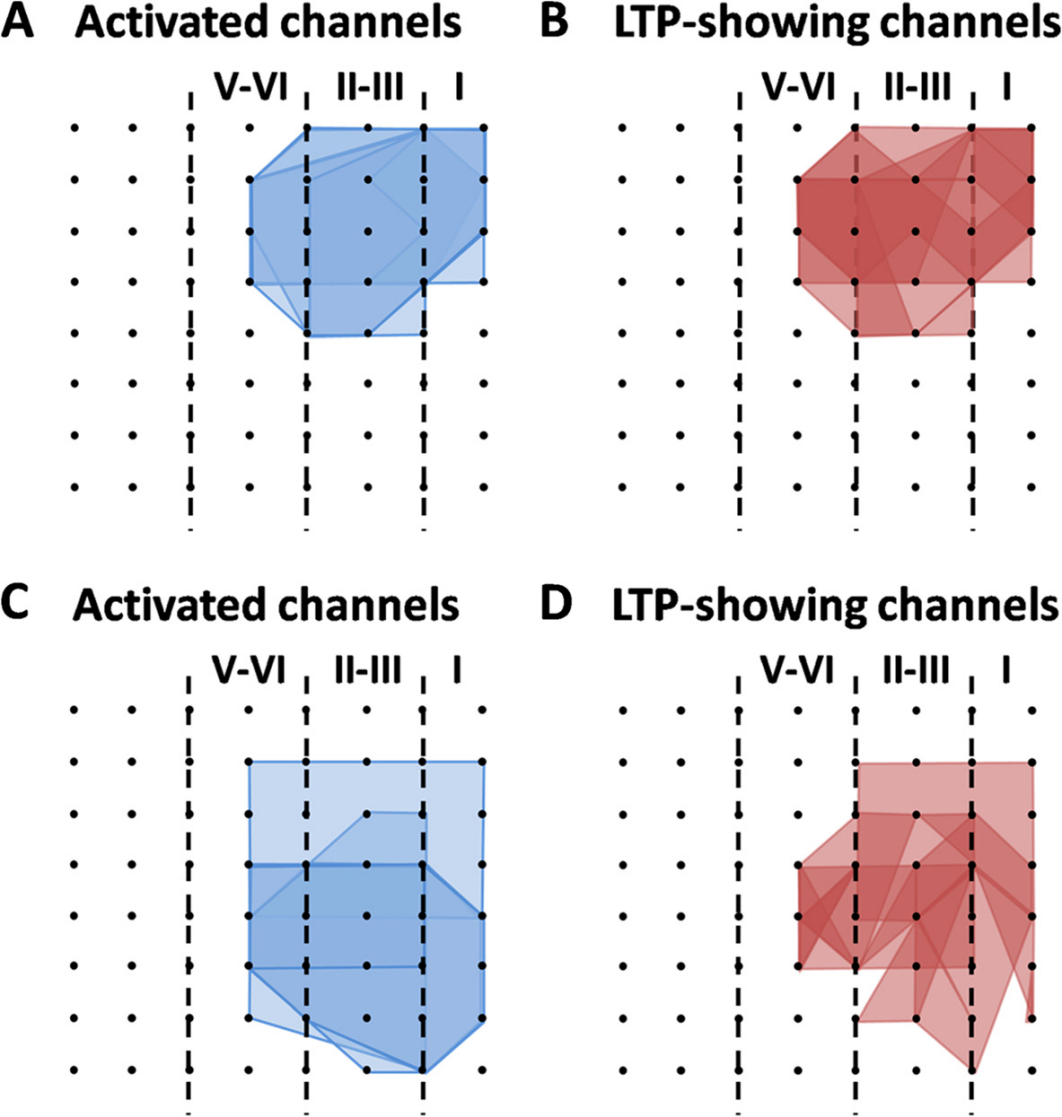
**Spatial analysis of LTP distribution in the ACC. (A and B)** Polygonal diagrams of the channels that were activated in the baseline (blue, **A**) and that showed LTP (red, **B**) in 6 slices from 6 mice. Vertical lines denote the specific layers in the ACC slice. Overlapped blue regions denote frequently activated channels, while overlapped red regions indicate the channels that are most likely to undergo LTP. **(C and D)** Similar as **(A and B)** but with TBS delivered in the presence of AMG9810 (n = 6 slices/6 mice). It is evident that the spatial distribution of LTP-showing channels is not altered by the TRPV1 antagonism.

In addition to the long-term recording of LTP across the extended time scale, 64-channel multisite recordings in the acute slice preparation can also allow us to perform spatial analysis of LTP distribution among the cortical network [64]. Therefore, we next analyzed the effect of AMG9810 on the spatial properties of LTP in the ACC. The number of activated channels and LTP-occurring channels was counted for each slice and then displayed by plotting a polygonal graph on a grid representing the 8 × 8 electrodes. We used the blue lines to represent the activated channels and the red lines to denote the LTP-showing channels as previously described [64]. Figure 2A and B show the grouped data of the control group (A, activated map; B, LTP map). We found that LTP cannot be induced in every channel of the ACC slice. In total, 86 channels (mean ± SEM: 14.3 ± 1.2) exhibited clear synaptic responses from 6 slices, with 71 channels (mean ± SEM: 11.8 ± 1.2) undergoing LTP. The remaining 15 channels exhibited short-term potentiation or remained unchanged at the baseline level after TBS. Bath application of AMG9810 (10 μM) did not affect the activation map nor the LTP map in the ACC (Figure 2C and D). Totally, 90 channels (mean ± SEM: 15.1 ± 1.2) were activated from 6 slices, and 69 channels (mean ± SEM: 11.6 ± 1.4) underwent LTP. By calculating the induction ratio of LTP (see Materials and methods), we did not observe any significant difference between control and AMG9810-treated group (control vs. AMG9810: 82.5 ± 4.4% vs. 76.7 ± 9.9%). Taken together, these findings suggest that pharmacological blockage of TRPV1 with AMG9810 could not prevent the induction of LTP in the ACC.

### SB366791, another TRPV1 antagonist, also has no effect on cingulate LTP induction

**Figure 3:**
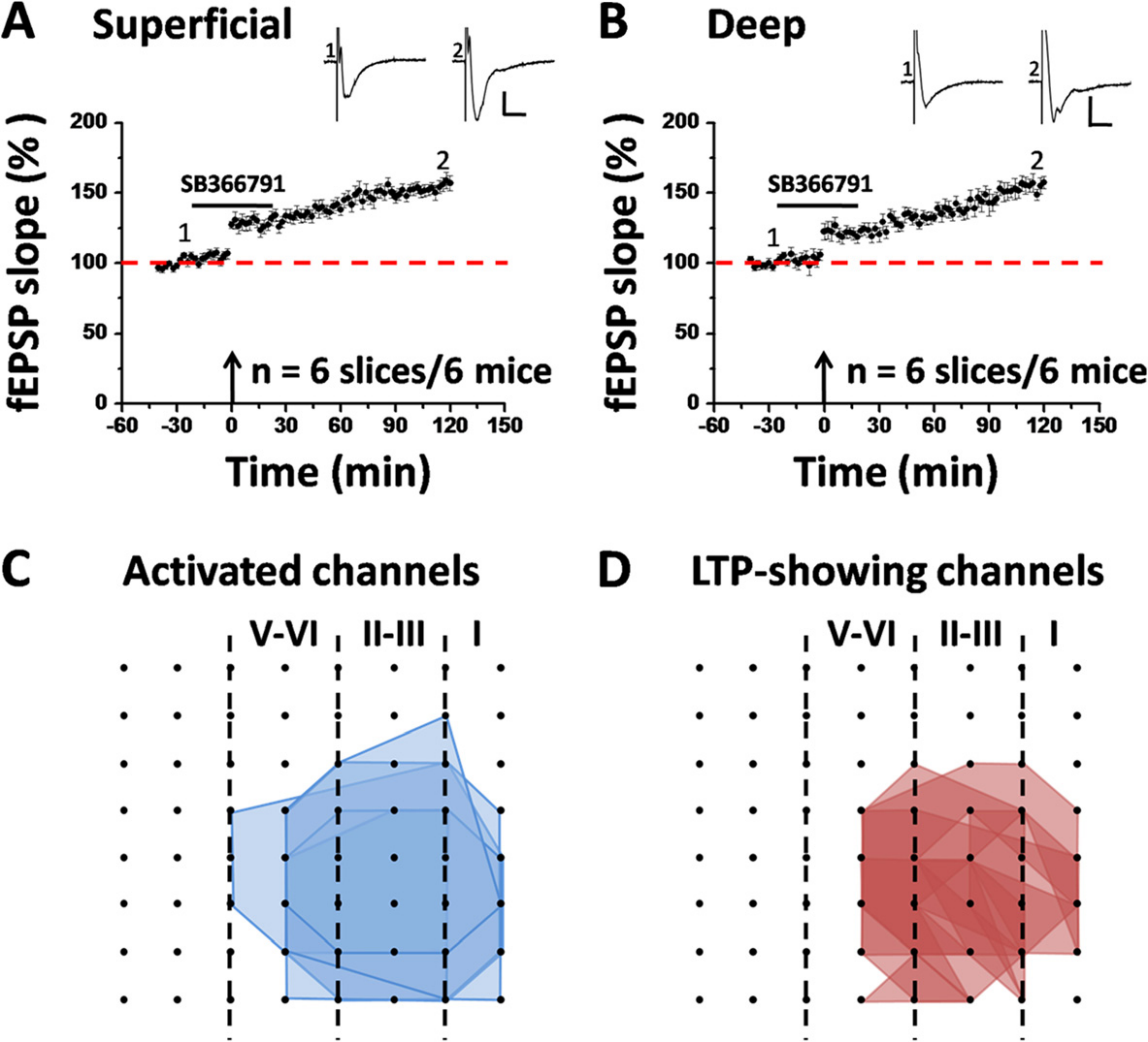
**SB366791 cannot block the induction of LTP in the adult mice ACC. (A)** Grouped data from 6 slices of 6 mice for SB366791 (20 μM), showing the normal induction of LTP in the superficial layer. **(B)** Summarized data in the deep layer (n = 6 slices/6 mice). Sample fEPSP recordings taken at the times indicated by the corresponding numbers are shown at the top of each plot. Arrows in **(A and B)** show the starting point of TBS application. Horizontal bars denote the period of drug delivery. Calibration: 100 μV, 10 ms. Error bars represent SEM. **(C and D)** Spatial analysis of the effect of SB366791 on LTP distribution in the ACC. Shown are polygonal graphs of the channels that were activated (blue, **C**) and that exhibited LTP (red, **D**) when TBS was delivered in the presence of SB366791 (n = 6 slices/6 mice). Vertical lines denote the specific layers in the ACC slice. SB366791 has no effect on the LTP distribution map in the ACC.

To confirm the above results, we performed pharmacological experiments using SB366791 (20 μM), another TRPV1 antagonist with high potency and an improved selectivity profile [66]. Pre-treatment of the ACC slice with SB366791 failed to produce any blocking effect on the induction of cingulate LTP in both superficial layer (156.9 ± 5.1% of baseline at 2 h after TBS, n = 6 slices/6 mice, *P* < 0.001, paired t-test, Figure 3A) and deep layer (157.3 ± 4.2% of baseline at 2 h after TBS, n = 6 slices/6 mice, *P* < 0.001, paired t-test, Figure 3B). Statistical analysis did not reveal any significant difference in the LTP extent between the two groups (control vs. SB366791: superficial layer, *P* = 0.070; deep layer, *P* = 0.671, unpaired t-test). We also determined the spatial distribution of LTP-occurring channels in the ACC with the presence of SB366791. Among the 6 slices analyzed, 108 channels (mean ± SEM: 18.6 ± 1.9) were activated and thus showing typical fEPSP in the baseline condition, while 84 channels (mean ± SEM: 14.4 ± 1.7) displayed LTP lasting for 2 h, with an induction ratio being 77.8 ± 7.2%. Thus, application of SB366791 could not alter the magnitude of cingulate LTP either.

### Pharmacological blockade of TRPV1 has no effect on LTD induction in the ACC

**Figure 4:**
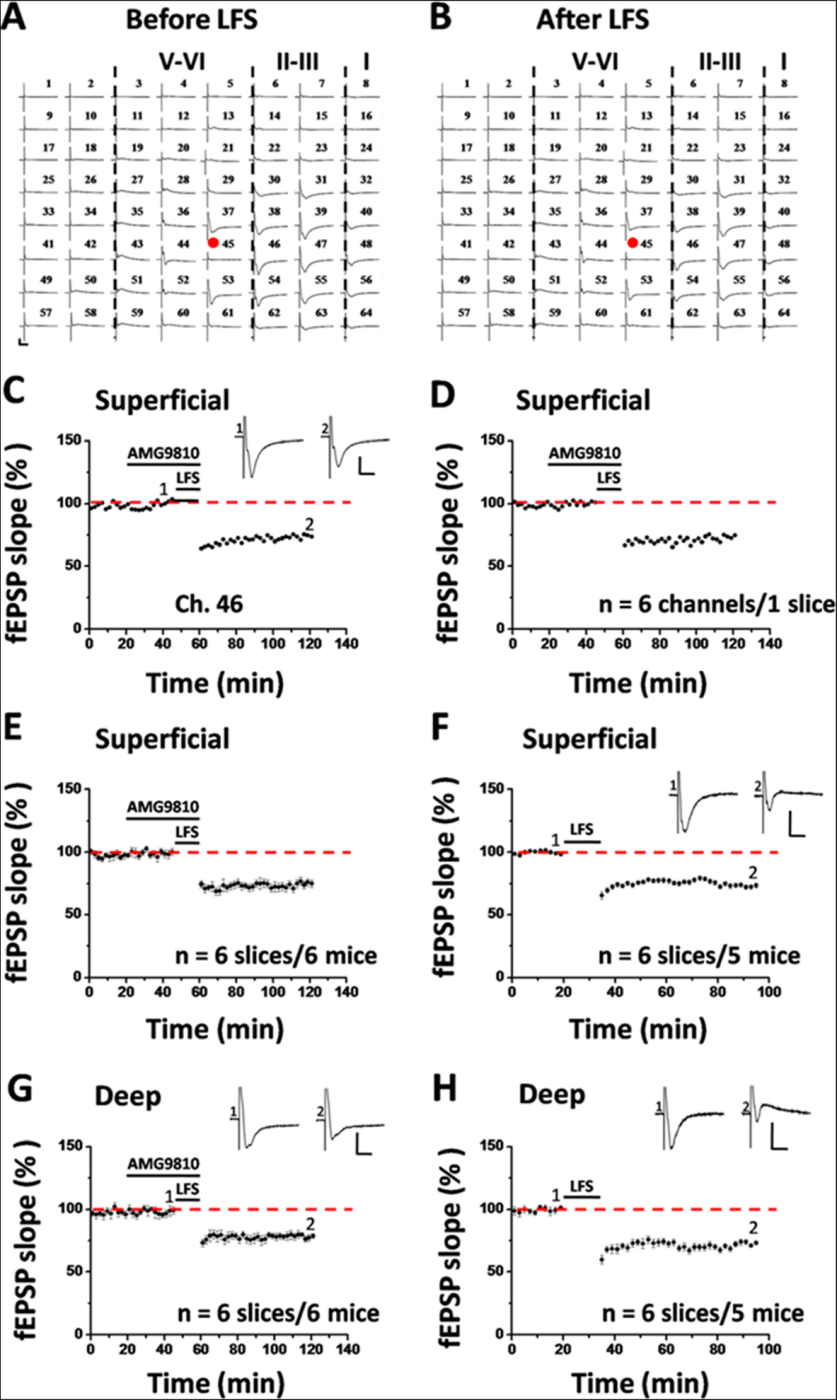
**Pharmacological blockade of TRPV1 activation with AMG9810 does not affect the LTD induction in the ACC. (A and B)** One sample of 64-channel recordings of ACC LTD induction in the presence of AMG9810 (10 μM). **A**, baseline; **B**, 1 h after LFS. AMG9810 was bath applied 25 min prior to and during the LFS delivery to the deep layer of the ACC slice. It is clearly illustrated that AMG9810 did not block LFS-induced LTD. Red circles indicate the stimulated channel (Ch. 45). Vertical lines demarcate the different layers in the ACC. **(C)** Results of one channel (Ch. 46) in the superficial layer of one ACC slice, showing the induction of LTD despite the presence of AMG9810. **(D)** Summary of averaged data from 6 superficial channels of the same slice. **(E)** Pooled data for the superficial layer from 6 slices from 6 mice. **(F)** Pooled data for the superficial layer of the ACC from control group (n = 6 slices/5 mice). **(G and H)** Summarized data for the deep layer of the ACC from control (**H**, n = 6 slices/5 mice) and AMG9810-treated (**G**, n = 6 slices/6 mice) group. Application of AMG9810 did not affect the induction of LTD in any layer of the ACC. Insets in **(C, F, G and H)**: representative fEPSP traces at time points indicated by the numbers in the graph. Horizontal bars in **(C-H)** denote the period of LFS or drug application as indicated. Calibrations in **(A, C, F, G and H)**: 100 μV, 10 ms. Error bars in **(E-H)** represent SEM.

Besides LTP, LTD is another important form of synaptic plasticity in the central nervous system [70,71]. Accumulating evidence has been presented to support the involvement of TRPV1 in various forms of LTD in the brain [24-26,32,33]. Our previous work has mapped the spatiotemporal properties of LTD in the ACC of adult mice [63]. Here, we evaluated the consequence of TRPV1 blockade on the induction probability of ACC LTD. One typical 64-channel recording of LTD in the presence of AMG9810 (10 μM) is illustrated in Figure 4A (before LFS) and Figure 4B (1 h after LFS). Figure 4C and D show one single channel (Ch. 46, 73.4% of baseline at 1 h after LFS) data and averaged results of 6 channels in the superficial layer from one slice (74.6% of baseline), respectively. Pooled data from a series of similar experiments are summarized in Figure 4E. Bath infusion of AMG9810 had no effect on the induction of LTD in the ACC (74.7 ± 2.8% of baseline, n = 6 slices/6 mice, *P* < 0.001, paired t-test), in comparison with the control group (73.4 ± 1.8% of baseline, n = 6 slices/5 mice, *P* < 0.001, paired t-test, Figure 4F). The lack of effect of AMG9810 on ACC LTD is also replicated in the deep layer of the ACC (control: 73.2 ± 1.4% of baseline, n = 6 slices/5 mice, *P* < 0.001, paired t-test, Figure 4H; AMG9810: 78.7 ± 1.8% of baseline, n = 6 slices/6 mice, *P =* 0.001, paired t-test, Figure 4G). The magnitude and duration of LFS-evoked LTD did not differ between the two groups (superficial layer: *P* = 0.878, unpaired t-test; deep layer: *P* = 0.065, Mann–Whitney rank sum test).

**Figure 5:**
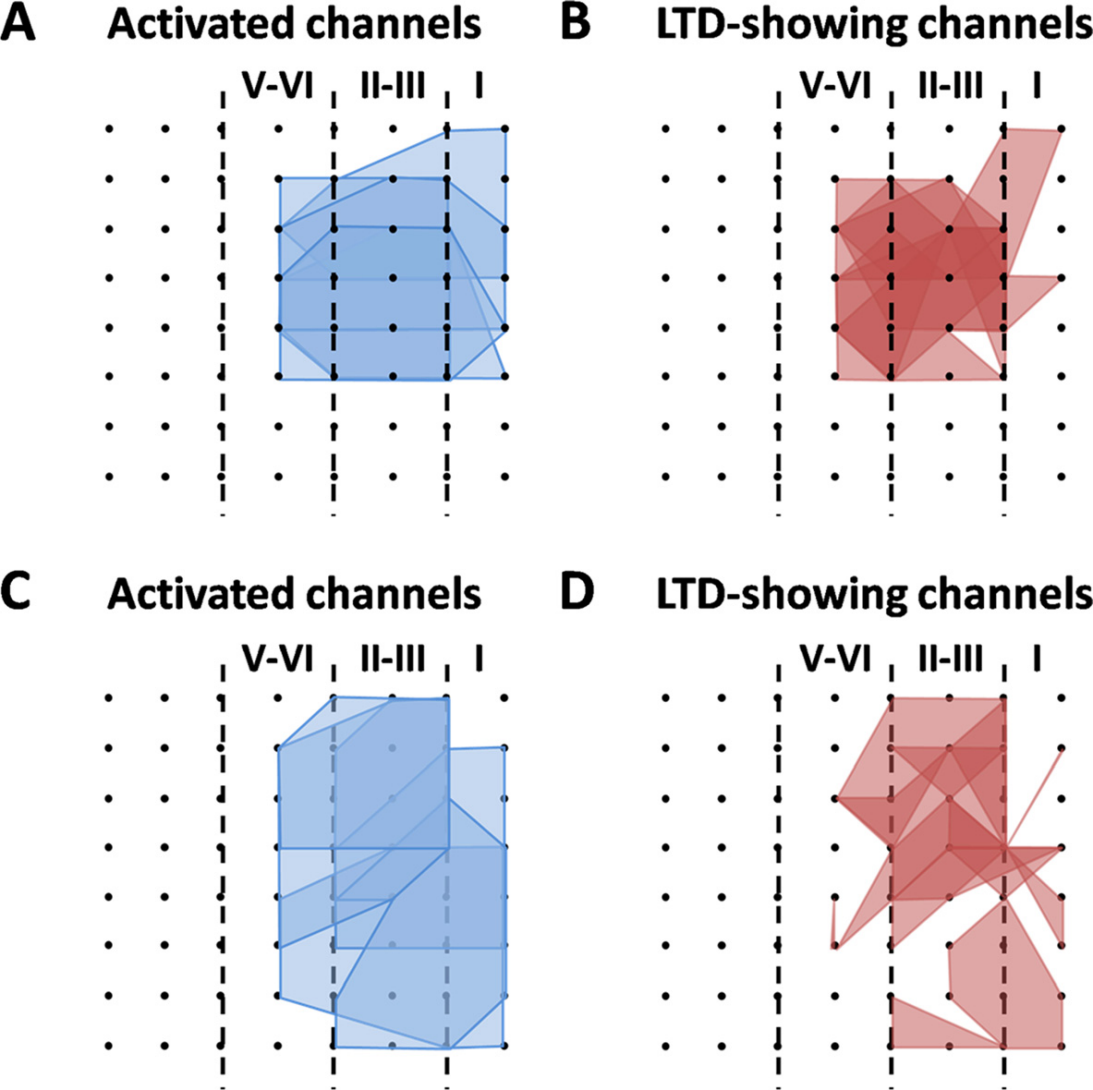
**Spatial analysis of LTD distribution in the ACC. (A and B)** Polygonal diagrams of the channels that were activated in the baseline (blue, **A**) and that showed LTD (red, **B**) in 6 slices from 5 mice. Vertical lines denote the specific layers in the ACC slice. Overlapped blue regions denote frequently activated channels, while overlapped red regions indicate the channels that are most likely to undergo LTD. **(C and D)** Similar as **(A and B)** but with LFS delivered in the presence of AMG9810 (n = 6 slices/6 mice). It is evident that the spatial distribution of LTD-showing channels is not altered by the TRPV1 antagonism.

Figure 5 demonstrates the spatial distribution maps of activated channels (A and C) and LTD-showing channels (B and D) for the control (A and B) and AMG9810-treated group (C and D). Consistent with our previous publication [63], LFS could not elicit LTD in every activated channel. There are 59 LTD-undergoing channels (mean ± SEM: 9.8 ± 0.2) out of 82 fEPSP-showing channels (mean ± SEM: 13.7 ± 0.8) in the control slices (induction ratio: 72.9 ± 3.4%, n = 6 slices/5 mice). In the AMG9810-applied slices, 93 channels (mean ± SEM: 15.5 ± 1.3) were activated, among which 66 channels (mean ± SEM: 10.9 ± 1.1) exhibited LTD (induction ratio: 71.0 ± 4.0%, n = 6 slices/6 mice).

**Figure 6:**
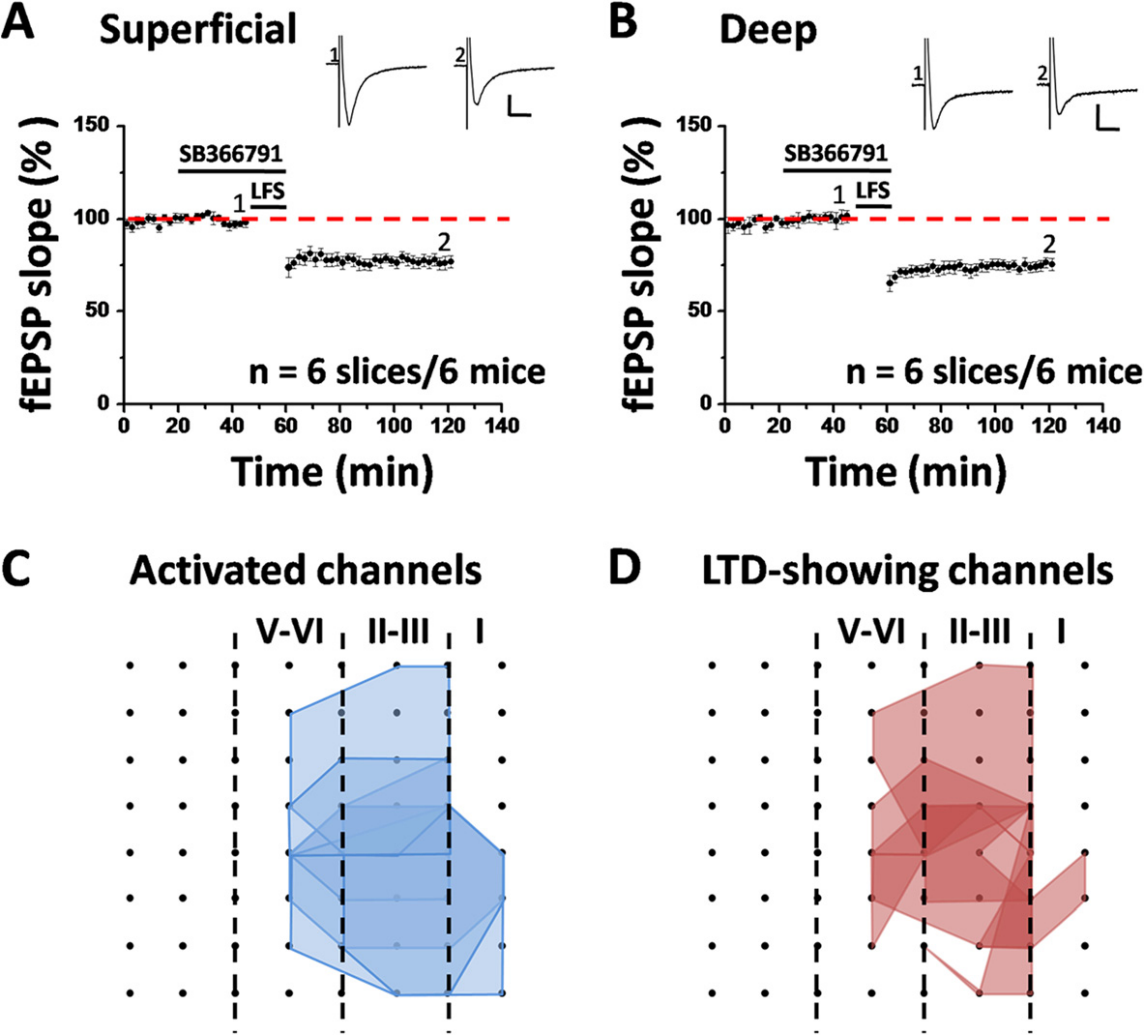
**SB366791 cannot block the induction of LTD in the adult mice ACC. (A)** Grouped data from 6 slices of 6 mice for SB366791 (20 μM), showing the normal induction of LTD in the superficial layer. **(B)** Summarized data in the deep layer (n = 6 slices/6 mice). Sample fEPSP recordings taken at the times indicated by the corresponding numbers are shown at the top of each plot. Horizontal bars denote the period of LFS or drug application as indicated. Calibration: 100 μV, 10 ms. Error bars represent SEM. **(C and D)** Spatial analysis of the effect of SB366791 on LTD distribution in the ACC. Shown are polygonal graphs of the channels that were activated (blue, **C**) and that exhibited LTD (red, **D**) when LFS was delivered in the presence of SB366791 (n = 6 slices/6 mice). Vertical lines denote the specific layers in the ACC slice. SB366791 has no effect on the LTD distribution map in the ACC.

Finally, we also tested the effect of SB366791 (20 μM) on LTD induction in the ACC. Delivery of LFS to the deep layer of the ACC slice still resulted in an enduring synaptic depression in the presence of SB366791. Specifically, the synaptic responses were depressed to 76.9 ± 3.3% of baseline (n = 6 slices/6 mice, *P* < 0.001, paired t-test) for the superficial layer (Figure 6A) and to 75.4 ± 3.3% (n = 6 slices/6 mice, *P* < 0.001, paired t-test) of baseline for the deep layer (Figure 6B) at 1 h after LFS. No significance was detected in the magnitude of LTD between control and SB366791-treated group (superficial layer: *P* = 0.959; deep layer: *P* = 0.462; unpaired t-test). Furthermore, the spatial distribution of LTD-showing channels was unaltered by SB366791 application (induction ratio: 69.6 ± 8.8%, n = 6 slices/6 mice, Figure 6C and D). Combining the data of AMG9810 and SB366791 together, it could be concluded that pharmacological blockade of TRPV1 channel activation cannot block LFS-induced LTD in the ACC.

## Discussion

In the present study, we used a 64-chanel multi-electrode array recording system to investigate the effect of two TRPV1 antagonists on LTP and LTD in the ACC of adult mice. Although the role of TRPV1 in synaptic plasticity has been reported in other brain areas (Table 2), we found that TRPV1 has no critical involvement in either TBS-induced LTP or LFS-induced LTD in the ACC.

### Distribution of TRPV1 in the brain

Since its first clone and characterization in 1997 [2], TRPV1 channels have been increasingly recognized to act as a molecular sensor for a range of noxious stimuli (proton, heat, and capsaicin) in the periphery [1,4,72]. Modulation of the expression or phosphorylation of TRPV1 in primary sensory neurons is believed to be an important mechanism underlying the development of thermal hyperalgesia in inflammatory conditions [8,73-75]. Therefore, pharmacological antagonism or genetic deletion of TRPV1 resulted in significant analgesic or anti-hyperalgesic effects in the preclinical research [5,6,11-13,67]. However, most of these previous studies have focused on the role of TRPV1 in pain processing at peripheral and spinal levels, with much less emphasis placed on the supraspinal or cortical level [59,76-78]. One of the major reasons for this phenomenon might be due to the early report showing the failure to detect TRPV1 mRNA or protein in the brain [1,2,27]. Nevertheless, a growing number of reports have indicated that TRPV1 is widely distributed in the central nervous system, covering a broad array of pain-related brain areas, such as somatosensory cortex, insular cortex, hippocampus, amygdala, thalamus and periaqueductal gray [14-17,28-30], Table 1]. In particular, it has also been shown to be expressed in the ACC [16,17,79], one of the most critical forebrain regions contributing to the pain perception [39,80,81]. Moreover, the brain function of TRPV1 has been gradually discovered, including control of body temperature, blood pressure, emesis, locomotion, anxiety and pain modulation [16]. There is also substantial evidence suggesting that TRPV1 mediates certain forms of synaptic plasticity in multiple brain regions [20], see below for details]. Taken together, all of the above descriptions highlight the importance of extending the TRPV1 research from periphery to the central nervous system.

### TRPV1 and LTP

As mentioned above, one of the emerging roles of TRPV1 in the brain is to mediate or facilitate LTP induction in the hippocampus (Table 2). Previous genetic studies demonstrated an attenuated pyramidal cell LTP in hippocampal CA1 region in the TRPV1 knock-out mice compared to the wild-type littermates [21-23]. This reduced LTP is thought to underlie the behavioral impairments in conditioned/cued fear memory, spatial memory and stress sensitization revealed in the TRPV1 deficient mice [21,22]. In addition, pharmacological activation of TRPV1 receptor by capsaicin or resiniferatoxin has been shown to facilitate the CA1 LTP induction by high-frequency stimulation and thus effectively prevent the blocking effect of stress on LTP in the hippocampus [22]. TBS-induced LTP could also be enhanced by TRPV1 agonists, which involves the GABAergic system in the hippocampal circuitry [31]. In contrast to the data in the hippocampus, the present results showed that pharmacological blockade of TRPV1 with either AMG9810 or SB366791 failed to affect the induction probability and magnitude of LTP in the ACC (Figures 1 and 3). The spatial distribution of LTP-showing channels among the ACC network was also unaltered by bath application of the TRPV1 antagonist (Figures 2 and 3), suggesting no critical involvement of TRPV1 in cingulate LTP. These negative results are likely not due to the insufficient drug dose applied during the experiment, because previous studies using the same dose or even lower dose of the drug got the positive results in vitro [22,25,26,35,82]. Instead, these data indicate that endogenous activation of TRPV1 is not important for TBS-induced LTP in the ACC.

### TRPV1 and LTD

In addition to LTP, evidence has also been provided to support the involvement of TPRV1 in various forms of LTD induction in the brain (Table 2). For example, TRPV1 can mediate a presynaptic form of LTD at glutamatergic synapses on hippocampal GABAergic interneurons, as revealed by both pharmacological and genetic approaches in both rats and mice [23,24,32]. On the other hand, treatment with TRPV1 agonists suppressed low-frequency stimulation-evoked LTD as well as (RS)-3,5-dihydroxyphenyl-glycine-induced acute depression in the CA1 region [22,31]. Apart from the hippocampus, TRPV1 activation by endocannabinoid contributes to the induction of a postsynaptic form of LTD in either dentate gyrus [25] or nucleus accumbens [26] or bed nucleus of the stria terminalis [34]. Intriguingly, we did not observe any blockade of LFS-evoked LTD by AMG9810 or SB366791 in the ACC slice of adult mice in the present study (Figures 4 and 6). Consistently, spatial network analysis failed to detect any difference in the LTD distribution map between control and drug-applied group (Figures 5 and 6). The reasons for the discrepancy are not clear but might be attributable to differences in the recording method, the specific TRPV1-targeting drug used and/or the brain regions studied. Overall, it seems likely that TRPV1 channel is not involved in either LTP or LTD induction in the ACC. However, previous studies have shown the expression of TRPV1 protein in the ACC via [^3^H] RTX binding assay [17] or immunohistochemistry [79]. Thus, TRPV1 channel may have a not yet identified role in the ACC other than mediating certain forms of synaptic plasticity.

### TRPV1 and chronic pain

As introduced above, the TRPV1 receptor is now considered a promising target for the development of novel analgesics with clinical potentials [57-61]. However, compared to the primary sensory neurons and spinal cord, much less is known about the function of TRPV1 in the brain [16,83]. The ACC is well known to be an important forebrain area that is critical for pain perception, modulation and chronic pain processing [80,84-88]. Our previous series of work have obtained the conclusion that synaptic plasticity in the ACC, like LTP and LTD, can serve as cellular model for studying chronic pain [39,42-46]. In the current study, we cannot detect any significant effect of TRPV1 antagonism on the induction of LTP or LTD in the ACC. This result indicates that TRPV1 antagonists could not block pain-related cortical plasticity. Consistent with the present study, the early work in TRPV1 knock-out mice showed the impaired behavioral responses to noxious heat stimuli and reduced acute inflammatory thermal hyperalgesia, but without any alteration in mechanical allodynia and neuropathic pain hypersensitivity [5,6]. Therefore, it is unlikely that any novel TRPV1 antagonists will be effective in the treatment of chronic inflammatory or neuropathic pain. Moreover, treating chronic pain patients with TRPV1 antagonists may produce untoward side effects, such as hyperthermia, increased susceptibility to cardiovascular diseases, etc. [60,61,89]. Possible side effects on cognitive and executive functions remain to be investigated, considering the important roles of TRPV1 in hippocampal plasticity as well as other regions of the brain.

## Competing interests

The authors declare that they have no competing interests.

## Authors’ contributions

M-GL performed the experiments, analyzed data and drafted the manuscript; MZ conceived and designed the research and finished the final version of the manuscript. Both authors read and approved the final manuscript.
